# Type 2 Diabetes Is Associated with Increased Coagulation Activity in Patients with Atrial Fibrillation: A D-Dimer-Based Analysis

**DOI:** 10.3390/biomedicines14020332

**Published:** 2026-01-31

**Authors:** Paul Gabriel Ciubotaru, Amit Kohli, Nilima Rajpal Kundnani, Roxana Buzas, Marioara Nicula Neagu, Marius Preda, Vlad-Sabin Ivan, Mihaela-Diana Popa, Milan Daniel Velimirovici, Daniel Florin Lighezan

**Affiliations:** 1 Department V, Internal Medicine I—Discipline of Medical Semiology I, Center of Advanced Research in Cardiology and Hemostasology, “Victor Babes” University of Medicine and Pharmacy, 300041 Timisoara, Romania; 2 Department of Anaesthesiology, Maulana Azad Medical College, New Delhi 110002, India; 3 University Clinic of Internal Medicine and Ambulatory Care, Prevention and Cardiovascular Recovery, Department VI-Cardiology, “Victor Babes” University of Medicine and Pharmacy, 3000041 Timisoara, Romania; 4 Research Centre of Timisoara, Institute of Cardiovascular Diseases, “Victor Babes” University of Medicine and Pharmacy, 3000041 Timisoara, Romania; 5 Physiology Discipline, Faculty of Bioengineering of Animal Resources, University of Life Sciences “King Mihai I” from Timisoara, 300645 Timisoara, Romania; 6 Second Discipline of Surgical Semiology, Department IX-Surgery-1, “Victor Babes” University of Medicine and Pharmacy, 300041 Timisoara, Romania; 7 Second Clinic of General Surgery and Surgical Oncology, Emergency Clinical Municipal Hospital, 300079 Timisoara, Romania; 8 Breast Surgery Research Center, “Victor Babes” University of Medicine and Pharmacy, 300041 Timisoara, Romania; 9 Department of Microbiology, “Victor Babes” University of Medicine and Pharmacy, 300041 Timisoara, Romania; 10 University Clinic of Practical Abilities, Department 1–Nursing, “Victor Babes” University of Medicine and Pharmacy, 300041 Timisoara, Romania

**Keywords:** D-dimer, heart failure, stroke, cardiometabolic risk factors, CHA_2_DS_2_-VASc score

## Abstract

**Background:** Atrial Fibrillation (AF) is associated with a prothrombotic state and increased risk of ischemic stroke. Type 2 diabetes mellitus (T2DM) is a major cardiometabolic comorbidity in AF and independently increases thromboembolic risk. D-dimer is a well-established biomarker of coagulation activation and fibrin turnover, but the specific contribution of T2DM to D-dimer levels in AF remains insufficiently characterized in real-world cohorts. **Methods:** We conducted a retrospective, observational, single-center study including 300 adult patients with non-valvular AF evaluated at a tertiary university hospital. Patients were stratified according to the presence of T2DM (150 with T2DM and 150 without diabetes). Plasma D-dimer levels were compared between groups and analyzed across clinically relevant thresholds and CHA_2_DS_2_-VASc categories. Multivariable linear and logistic regression models were used to assess the independent association between T2DM and D-dimer levels after adjustment for demographic factors, comorbidities, renal function, prior stroke, CHA_2_DS_2_-VASc score components, and oral anticoagulation. **Results:** Patients with T2DM exhibited significantly higher D-dimer levels than non-diabetic patients (median 0.94 vs. 0.63 µg/mL FEU, *p* < 0.001). T2DM was independently associated with higher log-transformed D-dimer levels (adjusted β = 0.19, *p* < 0.001) and with increased odds of elevated D-dimer above both 0.5 µg/mL and 1.0 µg/mL thresholds. Across all CHA_2_DS_2_-VASc categories, patients with T2DM consistently showed higher D-dimer concentrations. Findings remained robust in sensitivity analyses restricted to anticoagulated patients. **Conclusions:** In patients with atrial fibrillation, type 2 diabetes mellitus is associated with increased coagulation activity as reflected by higher D-dimer levels, independent of clinical thromboembolic risk. These results support the concept of a diabetes-associated hypercoagulable AF phenotype and highlight the potential role of coagulation biomarkers in refining risk stratification.

## 1. Introduction

Atrial Fibrillation (AF) is the most common sustained arrhythmia worldwide, affecting an estimated 40–60 million people, and its prevalence continues to rise due to population ageing and increasing cardiometabolic risk factors [1]. AF is associated with excess mortality, heart failure, hospitalizations and impaired quality of life, and has therefore become a major public health problem [2].

One of the most feared complications of AF is ischemic stroke. AF increases stroke risk approximately five-fold, and AF-related strokes tend to be more severe, with higher rates of disability and death than strokes unrelated to AF [3,4,5]. Contemporary European Society of Cardiology (ESC) guidelines [6] recommend that decisions on oral anticoagulation in patients with non-valvular AF are primarily based on the CHA_2_DS_2_-VASc score, which integrates congestive heart failure, hypertension, age, prior stroke or transient ischemic attack, vascular disease, sex, and diabetes mellitus into an easy-to-use risk stratification tool [7]. This clinical score has been validated in large real-world cohorts and remains the cornerstone of thromboembolic risk assessment in AF [7].

Beyond traditional clinical factors, circulating biomarkers have been extensively investigated as potential adjuncts to improve risk prediction in AF [8,9]. Among them, D-dimer, a degradation product of cross-linked fibrin, is a well-established marker of coagulation activation and secondary fibrinolysis [10,11]. D-dimer testing is routinely used in diagnostic algorithms for venous thromboembolism and disseminated intravascular coagulation, and elevated levels are associated with incident cardiovascular events and mortality in various cardiovascular conditions [11].

Higher D-dimer predicts stroke, embolism, bleeding, and death in AF. In ARISTOTLE, baseline D-dimer independently predicted thromboembolic events, major bleeding, and mortality beyond clinical risk scores [12]. Similar findings have been reported in other randomized trial cohorts, such as RE-LY, where elevated D-dimer during anticoagulation was linked to a higher risk of stroke, cardiovascular death and major bleeding [13]. Observational studies in “real-world” AF populations also indicate that elevated D-dimer is associated with higher rates of thromboembolic and cardiovascular events and may refine risk stratification beyond CHADS_2_ or CHA_2_DS_2_-VASc [14]. Taken together, these data support the concept that D-dimer reflects an underlying prothrombotic milieu in AF that is only partly captured by clinical scores [15].

Type 2 diabetes mellitus (T2DM) is highly prevalent and strongly linked to cardiovascular morbidity and mortality [16]. Patients with T2DM have a markedly increased risk of coronary artery disease, stroke and venous thromboembolism compared with the general population [17,18,19]. A characteristic feature of T2DM is a prothrombotic state, driven by platelet hyperreactivity, endothelial dysfunction, increased levels of procoagulant factors and impaired fibrinolysis [20]. Experimental and clinical studies consistently show hyperfibrinogenemia, elevated factor VIII and von Willebrand factor, increased plasminogen activator inhibitor-1 (PAI-1), altered fibrin clot structure and hypofibrinolysis in patients with T2DM [21]. These abnormalities result in dense, lysis-resistant fibrin networks and contribute to persistent activation of coagulation and elevated D-dimer levels in diabetes [22].

Recent reviews highlight that T2DM disrupts the balance between coagulation and fibrinolysis, promoting platelet hyperreactivity, altered clotting factors, and hypofibrinolysis, and that this prothrombotic state is only partly corrected by standard cardiometabolic therapy [23]. Elevated D-dimer has been associated with incident cardiovascular events, microvascular complications and mortality in patients with diabetes, reinforcing its role as a marker of ongoing thrombin generation and fibrin turnover in this high-risk population [10,23,24].

AF and T2DM frequently coexist, with registries showing that ~25% of AF patients have diabetes, which independently predicts adverse cardiovascular events and higher mortality [25,26]. Mechanistically, AF-related blood stasis, atrial cardiomyopathy and endothelial dysfunction may interact with diabetes-related vascular inflammation, hypercoagulability and hypofibrinolysis, creating an especially thrombogenic phenotype. Reviews show that D-dimer is higher in AF patients with comorbidities such as hypertension, heart failure, and diabetes, suggesting cardiometabolic factors amplify coagulation activation [27].

Few studies have examined D-dimer in AF stratified by diabetes status. Whether T2DM defines a distinct hypercoagulable AF phenotype—particularly in real-world Central/Eastern European cohorts—remains unclear, with potential implications for risk stratification and biomarker-guided management [15,28].

We conducted a retrospective single-center study in a real-world AF cohort from a tertiary university hospital in western Romania, comparing D-dimer levels between patients with and without T2DM. We hypothesized higher D-dimer in diabetic AF patients even after adjustment for major confounders, and secondarily assessed high D-dimer thresholds and CHA_2_DS_2_-VASc–stratified patterns.

## 2. Materials and Methods

### 2.1. Setup

This is a retrospective, observational, single-center study conducted at the Municipal Emergency Hospital Timișoara, a tertiary university hospital affiliated with the “Victor Babeș” University of Medicine and Pharmacy, Timișoara, Romania. We identified all adult patients evaluated for atrial fibrillation (AF) in the cardiology wards or outpatient cardiology clinics between January 2019 and December 2021, and screened them for eligibility based on predefined criteria. Data were obtained from the institutional electronic medical record. Data were extracted by two independent investigators and discrepancies were resolved by consensus.

### 2.2. Ethical Considerations

The study was conducted in accordance with the ethical principles of the Declaration of Helsinki for medical research involving human subjects, as updated by the World Medical Association in 2013 [29]. The protocol was reviewed and approved by the Ethics Committee of the Municipal Emergency Hospital Timișoara under approval number in collaboration with the Ethics Committee of the “Victor Babeș” University of Medicine and Pharmacy (approval number: 1697, approval date 21 March 2022). Data handling complied with the European Union General Data Protection Regulation (GDPR) (Regulation (EU) 2016/679) [30]. At hospital admission, all patients signed a standardized institutional informed consent form that permits storage and secondary use of anonymized clinical and laboratory data for research and quality improvement. For this analysis, the dataset was fully anonymized prior to export; no direct identifiers (such as name, national identification number or address) were retained, and results are reported only in aggregated form.

### 2.3. Patient Selection

Adult patients aged 18 years or older with non-valvular atrial fibrillation were eligible for screening. AF was defined and classified according to the latest European Society of Cardiology (ESC) Guidelines for the diagnosis and management of atrial fibrillation [6]. We included patients if they had at least one quantitative plasma D-dimer measurement performed during hospital admission or outpatient evaluation and if key clinical data—particularly type 2 diabetes mellitus status, renal function and anticoagulation therapy at the time of sampling—were available.

Where documented, atrial fibrillation type (either persistent or permanent) was recorded; however, AF subtype was not consistently available in all records and was not included as a mandatory selection variable.

Patients were excluded if they had evidence of valvular AF, defined as the presence of mechanical prosthetic heart valves or moderate to severe rheumatic mitral stenosis in line with ESC guidance [6]. We also excluded patients with acute venous thromboembolism diagnosed at the index encounter, recent major surgery or significant trauma within the preceding four weeks, active malignancy under systemic oncologic treatment, or severe acute infection or systemic inflammatory disease, or a recent history of COVID-19 infection, at the time of D-dimer testing, because these conditions are known to cause marked and acute D-dimer elevations independent of chronic cardiometabolic status. In addition, patients with end-stage kidney disease requiring chronic dialysis and those with missing essential data on diabetes status or D-dimer values were excluded.

All eligible non-valvular AF patients within the study period were screened consecutively from the institutional electronic medical record. After applying predefined exclusion criteria, the final analytic cohort was constructed using a balanced design: all eligible patients with documented T2DM were included, and an equal number of non-diabetic AF patients were selected from the remaining eligible pool to achieve a 1:1 distribution. Non-diabetic patients were selected without reference to D-dimer values.

### 2.4. Variables Studied

For each patient, we recorded baseline age, sex and body mass index values.

T2DM was defined according to the American Diabetes Association (ADA) “Standards of Care in Diabetes” [31], which allow diagnosis based on any of the following: fasting plasma glucose ≥ 126 mg/dL (7.0 mmol/L), 2-h plasma glucose ≥ 200 mg/dL (11.1 mmol/L) during a 75 g oral glucose tolerance test, glycated hemoglobin (HbA1c) ≥ 6.5%, or random plasma glucose ≥ 200 mg/dL (11.1 mmol/L) in the presence of classic symptoms of hyperglycemia, confirmed by repeat testing in the absence of unequivocal hyperglycemia.

T2DM status was determined as a pre-existing diagnosis documented in the medical record and/or current use of glucose-lowering therapy prior to the index D-dimer measurement; incident hyperglycemia identified only at the time of the index encounter was not classified as established T2DM.

Where available, additional diabetes-related variables were extracted from the medical record, including diabetes duration, glycated hemoglobin (HbA1c), and antidiabetic therapy class (e.g., metformin, insulin, SGLT2 inhibitors, GLP-1 receptor agonists). However, these parameters were not consistently documented for the entire cohort and were therefore not included as adjustment variables in the primary multivariable models.

Hypertension diagnosis was based on criteria following the 2018 ESC/ESH Guidelines for the management of arterial hypertension [32], which define hypertension as persistent office blood pressure ≥ 140/90 mmHg confirmed on repeated measurements or out-of-office monitoring.

Heart failure was considered present when recorded by a cardiologist based on clinical assessment, imaging and laboratory data, in line with the ESC Guidelines for the diagnosis and treatment of acute and chronic heart failure [33], which require symptoms and/or signs of heart failure and objective evidence of cardiac structural or functional abnormality.

Chronic kidney disease (CKD) was defined as an estimated glomerular filtration rate (eGFR) < 60 mL/min/1.73 m^2^, consistent with the KDIGO CKD guideline [34]. Estimation of GFR was performed using the CKD-EPI equation implemented in the hospital laboratory.

Other comorbid conditions such as coronary artery disease, prior myocardial infarction, prior ischemic stroke or transient ischemic attack, peripheral arterial disease and chronic obstructive pulmonary disease were identified and noted.

Plasma D-dimer levels (µg/mL FEU) were measured in citrated venous blood using the hospital’s standardized quantitative immunoturbidimetric assay. To reduce biological variability related to timing and acute clinical fluctuations, we used the first D-dimer value obtained within the first 24 h of hospital admission. For patients evaluated in an outpatient setting, we used the D-dimer result closest in time to the cardiology consultation (same-day where available), and only stable, non-acute encounters were included according to the exclusion criteria. The assay reference range used by the institutional laboratory was <0.5 µg/mL FEU.

Where available, inflammatory markers such as C-reactive protein (mg/L) and hemoglobin levels (g/dL) were also extracted.

Thromboembolic and bleeding risk were quantified using the CHA_2_DS_2_-VASc and HAS-BLED scores. The CHA_2_DS_2_-VASc score was calculated according to the original, later validated, Birmingham schema [7,35], in which congestive heart failure, hypertension, age ≥ 75 years (2 points), diabetes mellitus, prior stroke or transient ischemic attack (2 points), vascular disease, age 65–74 years and female sex are assigned specific points to estimate stroke risk in non-valvular AF. The HAS-BLED score was calculated following the original, further Guideline-endorsed, Euro Heart Survey derivation [6,36], assigning one point each for hypertension, abnormal renal or liver function, stroke, bleeding history or predisposition, labile international normalized ratio, elderly age and concomitant drugs or alcohol.

Medication use at the time of D-dimer sampling was recorded, including type of oral anticoagulation (vitamin K antagonist, direct oral anticoagulant, or no anticoagulant), antiplatelet therapy, statins, beta-blockers, renin-angiotensin system inhibitors and antidiabetic drugs.

### 2.5. Study Design and Outcomes

For the main comparative analyses, we stratified the cohort into two groups according to diabetes status: AF patients with T2DM and AF patients without diabetes. The primary outcome variable was the plasma D-dimer concentration. Secondary outcomes included the prevalence of high D-dimer above a predefined threshold and the distribution of D-dimer levels across CHA_2_DS_2_-VASc categories, as well as the independent association between T2DM and elevated D-dimer after adjustment for potential confounders.

D-dimer thresholds of >0.5 µg/mL FEU and >1.0 µg/mL FEU were selected because 0.5 µg/mL FEU is locally used as a conventional clinical threshold in diagnostic and cardiovascular risk contexts, while 1.0 µg/mL FEU was used as a higher-risk cutoff to explore more pronounced coagulation activation and improve interpretability for clinical stratification.

### 2.6. Statistical Analysis

All statistical analyses were performed using IBM SPSS Statistics version 26 (IBM Corp., Armonk, NY, USA) and R software version 4.3.3 (R Foundation for Statistical Computing, Vienna, Austria). The distribution of continuous variables was assessed using the Shapiro–Wilk test and visual inspection of histograms and Q-Q plots. Normally distributed continuous variables were expressed as mean and standard deviation, whereas skewed variables were summarized as median and interquartile range. Categorical variables were expressed as absolute numbers and percentages. Between-group comparisons for continuous variables were performed using Student’s *t*-test or the Mann–Whitney U test, as appropriate. Categorical variables were compared using the chi-square test or Fisher’s exact test.

Because D-dimer values are typically right-skewed, we anticipated log-transformation of D-dimer (log_10_ D-dimer) for parametric analyses. Multivariable linear regression models were built with log-transformed D-dimer as the dependent variable to assess the independent association between T2DM and coagulation activity, adjusting for clinically relevant covariates including age, sex, heart failure, renal function, prior stroke or transient ischemic attack, CHA_2_DS_2_-VASc score components and oral anticoagulation. In complementary analyses, we constructed multivariable logistic regression models with high D-dimer (above each threshold) as the binary dependent variable and T2DM as the main independent variable, again adjusting for the same covariates. We performed prespecified sensitivity analyses by excluding patients not receiving any oral anticoagulant, by stratifying the cohort by sex, and by excluding extreme D-dimer outliers above a predefined percentile to assess the robustness of the findings. A two-sided *p* value < 0.05 was considered statistically significant.

Oral anticoagulation at the time of sampling was modeled as a categorical covariate (DOAC, VKA, or no oral anticoagulation).

To reduce multicollinearity, we avoided simultaneously including both the composite CHA_2_DS_2_-VASc score and its individual components within the same multivariable model. Multicollinearity was assessed using variance inflation factors (VIF), and covariates with evidence of redundancy were handled by selecting clinically representative variables rather than overlapping constructs.

Missing data were minimal for the main exposure (T2DM) and outcome (D-dimer). Analyses were performed using complete-case methods for each model, without imputation.

As a sensitivity analysis, we performed propensity score matching (PSM) to minimize baseline differences between groups. Propensity scores were estimated using logistic regression with T2DM status as the dependent variable and age and renal function (eGFR) as matching covariates. We applied 1:1 nearest-neighbor matching without replacement using a caliper of 0.2 standard deviations of the logit of the propensity score. Balance was assessed using standardized mean differences.

## 3. Results

### 3.1. Patient Selection and Study Population

During the study period, a total of 412 adult patients with documented atrial fibrillation and at least one available D-dimer measurement was initially screened. After exclusion of patients with valvular atrial fibrillation (*n* = 21), acute venous thromboembolism at presentation (*n* = 28), recent major surgery or trauma (*n* = 19), active malignancy under systemic treatment (*n* = 24), severe acute infection or inflammatory disease (*n* = 17), end-stage renal disease requiring dialysis (*n* = 11), and incomplete data on diabetes status or D-dimer levels (*n* = 22), 300 patients were included in the final analysis.

**Figure 1:**
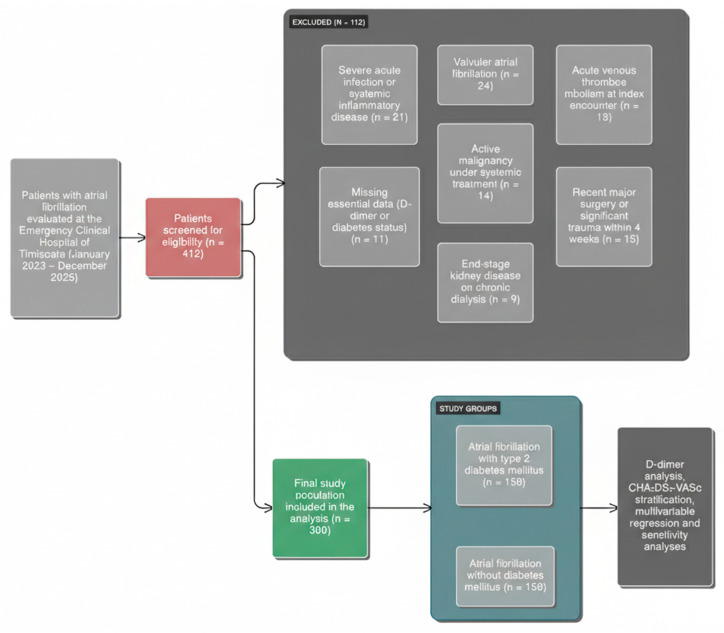
Patient Selection Flowchart. Patient selection and exclusion process for the retrospective analysis of atrial fibrillation patients. Of 412 screened patients, 112 were excluded based on predefined criteria, resulting in a final study population of 300 patients, stratified according to the presence or absence of type 2 diabetes mellitus.

Of these, 150 patients had a documented diagnosis of type 2 diabetes mellitus (T2DM), while 150 patients had no history of diabetes and were classified as non-diabetic. The patient selection process and reasons for exclusion are summarized in Figure 1.

### 3.2. Baseline Characteristics

Baseline demographic and clinical characteristics of the study population stratified by diabetes status are presented in Table 1. Patients with T2DM were significantly older than those without diabetes, with a mean age of 72.6 ± 8.9 years compared with 68.1 ± 10.4 years in the non-diabetic group (*p* < 0.001). Female sex distribution was similar between groups (46.7% in the T2DM group vs. 48.0% in the non-diabetic group, *p* = 0.82).

As expected, cardiometabolic comorbidity burden was higher among patients with T2DM. Hypertension was present in 86.0% of diabetic patients compared with 68.7% of non-diabetic patients (*p* < 0.001), and chronic kidney disease (eGFR < 60 mL/min/1.73 m^2^) was more frequent in the T2DM group (38.0% vs. 21.3%, *p* = 0.002). Heart failure was also more prevalent among patients with T2DM (44.7% vs. 29.3%, *p* = 0.006). Prior ischemic stroke or transient ischemic attack was documented in 22.0% of diabetic patients and 14.0% of non-diabetic patients (*p* = 0.08).

Consistent with these differences, the median CHA_2_DS_2_-VASc score was higher in patients with T2DM (4 (IQR 3–5)) than in those without diabetes (3 (IQR 2–4), *p* < 0.001). Oral anticoagulation at the time of D-dimer sampling was used in 81.3% of the overall cohort, with no significant difference between groups (T2DM: 82.7% vs. non-diabetic: 80.0%, *p* = 0.56). Direct oral anticoagulants were the most frequently prescribed agents in both groups.

### 3.3. D-Dimer Levels According to Diabetes Status

**Figure 2:**
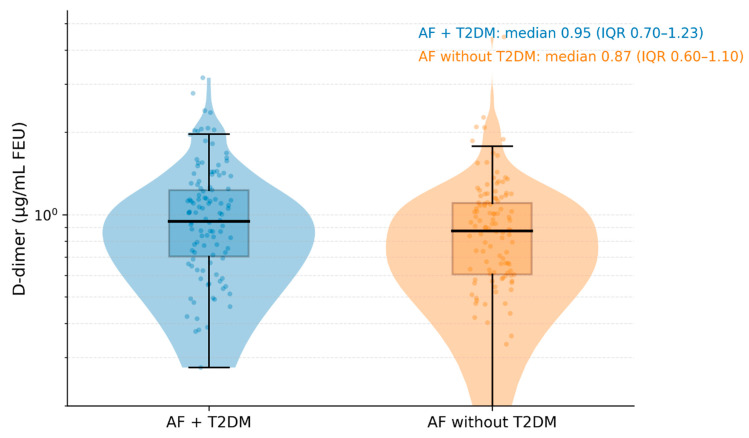
D-dimer levels according to diabetes status in patients with atrial fibrillation. Violin plots illustrate the distribution density of plasma D-dimer concentrations (µg/mL FEU) in patients with atrial fibrillation (AF) with type 2 diabetes mellitus (T2DM) and in those without diabetes. Overlaid boxplots depict the median (horizontal line), interquartile range (box), and whiskers extending to 1.5× the interquartile range. Jittered points represent individual patient values (subsampled for visual clarity). The *y*-axis is displayed on a logarithmic scale to account for the right-skewed distribution of D-dimer values typical of clinical cohorts.

Plasma D-dimer levels were right-skewed in both groups. AF patients with T2DM exhibited significantly higher D-dimer concentrations compared with those without diabetes. Median D-dimer was 0.94 µg/mL FEU (IQR 0.56–1.62) in the T2DM group versus 0.63 µg/mL FEU (IQR 0.38–1.05) in the non-diabetic group (*p* < 0.001). The distribution of D-dimer values by diabetes status is illustrated in Figure 2.

When analyzed on the logarithmic scale, as seen in Table 2, mean log_10_ (D-dimer) values were also higher among diabetic patients (−0.03 ± 0.48) compared with non-diabetic patients (−0.20 ± 0.44, *p* < 0.001).

### 3.4. Categorical D-Dimer Threshold Analyses

Using clinically relevant thresholds, a higher proportion of patients with T2DM demonstrated elevated D-dimer levels. A D-dimer value above 0.5 µg/mL FEU was observed in 74.0% of diabetic patients compared with 56.7% of non-diabetic patients (*p* = 0.002). Using a higher threshold of 1.0 µg/mL FEU, elevated D-dimer was present in 42.7% of patients with T2DM versus 26.0% of patients without diabetes (*p* = 0.003).

### 3.5. D-Dimer Levels Across CHA_2_DS_2_-VASc Categories

**Figure 3:**
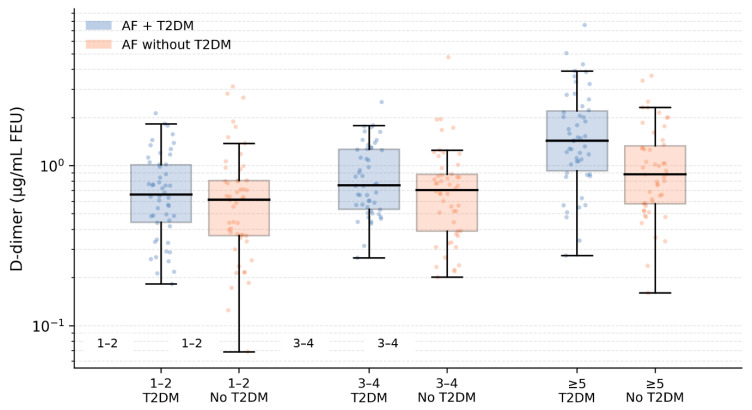
D-dimer levels across CHA_2_DS_2_-VASc categories. Plasma D-dimer concentrations (µg/mL FEU) are shown across increasing CHA_2_DS_2_-VASc score categories (1–2, 3–4, and ≥5) in patients with atrial fibrillation, stratified by the presence of type 2 diabetes mellitus (T2DM). Boxes represent the interquartile range, the central horizontal line indicates the median, and whiskers extend to 1.5× the interquartile range. Individual patient values are overlaid as jittered points to illustrate data distribution. The *y*-axis is displayed on a logarithmic scale due to the right-skewed distribution of D-dimer values. Across all CHA_2_DS_2_-VASc strata, patients with T2DM exhibit higher D-dimer levels than non-diabetic patients.

To further examine the relationship between coagulation activity, clinical thromboembolic risk, and diabetes status, D-dimer levels were analyzed across CHA_2_DS_2_-VASc score categories (Figure 3). In both groups, D-dimer concentrations increased progressively with higher CHA_2_DS_2_-VASc categories (*p* for trend < 0.001 for both). Among patients with CHA_2_DS_2_-VASc scores of 1–2, median D-dimer levels were higher in those with type 2 diabetes mellitus (0.65 µg/mL FEU (IQR 0.40–0.95)) compared with non-diabetic patients (0.48 µg/mL FEU [IQR 0.30–0.75], *p* = 0.012). Similar differences were observed in patients with CHA_2_DS_2_-VASc scores of 3–4 (0.92 (0.60–1.35) vs. 0.65 (0.42–1.00) µg/mL FEU, *p* = 0.004) and in those with scores ≥ 5 (1.35 (0.85–2.10) vs. 0.98 (0.60–1.55) µg/mL FEU, *p* = 0.018). These findings indicate that, within each level of clinical thromboembolic risk, patients with diabetes exhibit higher coagulation activity, suggesting an additive hypercoagulable effect of diabetes beyond CHA_2_DS_2_-VASc-based risk stratification.

### 3.6. Multivariable Analyses

In multivariable linear regression models using log-transformed D-dimer as the dependent variable, type 2 diabetes mellitus remained independently associated with higher D-dimer levels after adjustment for age, sex, heart failure, renal function, prior ischemic stroke/TIA, CHA_2_DS_2_-VASc score and oral anticoagulation at the time of sampling (adjusted β = 0.19, 95% CI 0.09–0.29, *p* < 0.001).

In multivariable logistic regression analyses, as seen in Table 3, T2DM was independently associated with an increased likelihood of elevated D-dimer. Specifically, T2DM was associated with higher odds of D-dimer > 0.5 µg/mL FEU (adjusted OR 2.18, 95% CI 1.34–3.55, *p* = 0.002) and D-dimer > 1.0 µg/mL FEU (adjusted OR 2.07, 95% CI 1.24–3.47, *p* = 0.005).

Clinically, this data corresponds to approximately a two-fold higher probability of elevated D-dimer in patients with T2DM compared with non-diabetic patients, even after accounting for age, renal function, heart failure, prior stroke/TIA, and anticoagulation status. This supports the concept of a diabetes-associated prothrombotic phenotype within atrial fibrillation.

### 3.7. Sensitivity Analyses and Propensity Score Matching

Sensitivity analyses yielded consistent results. When restricting the analysis to patients receiving oral anticoagulation, median D-dimer levels remained significantly higher in the T2DM group (0.89 (0.54–1.51) µg/mL FEU) compared with non-diabetic patients (0.61 (0.37–0.98) µg/mL FEU, *p* < 0.001). Exclusion of extreme D-dimer values above the 99th percentile did not materially alter the magnitude or direction of the association. In sex-stratified analyses, the association between T2DM and higher D-dimer persisted in both men and women, although statistical significance was attenuated in women due to smaller subgroup size.

In the propensity score-matched cohort (1:1 matching on age and eGFR), baseline differences between groups were reduced (standardized mean differences < 0.1 for matched covariates). D-dimer levels remained significantly higher in the T2DM group compared with matched non-diabetic patients, confirming the robustness of the primary findings.

## 4. Discussion

### 4.1. Principal Findings and Interpretation

In this real-world cohort of patients with atrial fibrillation, we observed a consistent association between type 2 diabetes mellitus and higher plasma D-dimer concentrations, suggesting increased ongoing coagulation activity in diabetic patients compared with non-diabetic patients. This finding is clinically and biologically coherent with the concept that AF is accompanied by a systemic prothrombotic milieu and that D-dimer integrates information about thrombin generation, fibrin formation, and secondary fibrinolysis. Early mechanistic work already showed that patients with AF exhibit elevated circulating markers of thrombogenesis, including fibrin D-dimer, even outside overt thromboembolic events, supporting the notion of “baseline” hypercoagulability in AF [37].

From a prognostic standpoint, D-dimer is not merely a nonspecific laboratory abnormality in AF [11]; rather, it has repeatedly been associated with major clinical outcomes. In the ARISTOTLE biomarker analysis, higher baseline D-dimer levels were linked to increased risks of stroke/systemic embolism, major bleeding, and mortality, and the addition of D-dimer improved the predictive performance of common clinical risk scores [12]. Similar findings have been reported in RE-LY, where baseline D-dimer was related to stroke/systemic embolism, cardiovascular death, and major bleeding, and where adding D-dimer improved risk prediction beyond established clinical factors [8,9]. Observational data also support the clinical relevance of D-dimer even during anticoagulation; for example, in non-valvular AF, D-dimer combined with clinical risk factors predicted subsequent thromboembolic events despite warfarin therapy [38]. Collectively, these studies establish D-dimer as a robust marker of residual risk and ongoing coagulation activation in AF, consistent with contemporary reviews of the biological and clinical evidence linking AF to hypercoagulability and fibrin turnover [39].

Within this established AF biomarker framework, the present results suggest that T2DM identifies a subset of AF patients with more pronounced activation of coagulation/fibrin turnover. This interpretation is strengthened by our CHA_2_DS_2_-VASc-stratified analysis: D-dimer increased in a stepwise fashion across higher CHA_2_DS_2_-VASc categories in both groups, supporting internal biological consistency between clinical risk gradients and coagulation activity. This aligns with the broader field’s move toward complementing clinical risk prediction with biomarkers: biomarker-based tools, such as the ABC-stroke score [8], can outperform purely clinical scores, underscoring that objective circulating markers capture risk information not fully encoded by comorbidity checklists.

Longer diabetes duration has been associated with higher thromboembolism risk in patients with AF, without a parallel increase in anticoagulant-related bleeding risk, suggesting a net shift toward thrombosis in long-standing diabetes [40,41]. In complementary datasets, diabetes duration has emerged as a stronger predictor of ischemic stroke than glycemic control metrics alone in AF populations, while other work suggests that both diabetes duration and HbA1c can contribute to thromboembolic risk gradients in incident AF with T2DM [40,42].

### 4.2. Comparison with Prior Biomarker Literature and What Is Novel in This Study

Atrial fibrillation is increasingly understood not only as an electrical disorder but also as a condition characterized by systemic changes in coagulation and fibrinolysis. Circulating biomarkers of coagulation activation, particularly D-dimer, have been extensively evaluated in AF cohorts for their prognostic significance [12,43,44]. D-dimer represents a fibrin degradation product that emerges during fibrinolysis after cross-linked fibrin formation, and elevated concentrations reflect active coagulation and subsequent clot breakdown [10,27]. Historically, D-dimer has been used clinically to rule out acute venous thromboembolism [45]; however, in the setting of AF it has prognostic relevance beyond diagnostic exclusion. In a large systematic review, numerous coagulation factors, including D-dimer, were associated with both prevalent and incident atrial fibrillation, reinforcing the idea that AF is a prothrombotic condition [9,46].

In a retrospective study, elevated D-dimer was independently associated with stroke risk in patients with non-valvular AF, emphasizing its potential as a prognostic biomarker beyond clinical risk factors alone [47]. Biomarker-enhanced risk prediction models, such as the ABC [8] (Age, Biomarkers, Clinical history) stroke risk score, have incorporated D-dimer alongside other markers to improve on clinical models like CHA_2_DS_2_-VASc [28], further underscoring the relevance of circulating coagulation markers in stratifying thromboembolic risk. Nonetheless, many of these studies examined D-dimer as part of multimarker panels or in relation to clinical outcomes, without specifically addressing how comorbid conditions such as diabetes modify D-dimer levels within AF populations.

The novelty of this study is the direct comparison of D-dimer levels in AF patients with versus without T2DM, a design rarely addressed in prior biomarker research. Although diabetes is a recognized stroke risk factor in AF and is included in the CHA_2_DS_2_-VASc score, its specific impact on a validated marker of coagulation activation has not been systematically quantified in AF cohorts. Our results show consistently higher D-dimer levels in diabetic AF patients across risk strata, providing a biological correlate to the established epidemiological risk associated with diabetes.

The distinct hypercoagulable phenotype that we describe aligns with work in non-AF populations showing altered coagulation dynamics in T2DM. Tripodi et al. demonstrated that patients with T2DM show an imbalance between pro- and anticoagulant pathways, leading to enhanced thrombin generation and elevated circulating pro-coagulant microparticles, even when conventional coagulation assays are normal [48]. These alterations in hematologic milieu plausibly contribute to the elevated D-dimer observed in diabetic individuals and may help explain the additive effect observed in our AF population.

Reviews show that diabetes promotes a prothrombotic state through increased platelet activation, higher procoagulant factors (e.g., fibrinogen, VII, VIII), and impaired fibrinolysis [49,50,51]. Platelet indices such as mean platelet volume and platelet distribution width, which can be elevated in T2DM, have also been associated with augmented thrombotic risk and adverse vascular outcomes [49,52,53].

Most prior AF biomarker studies linked D-dimer to overall risk without stratifying by diabetes, typically treating T2DM as a covariate rather than the main exposure [54]. In contrast, our study explicitly positions diabetes as a determinant of coagulation activation within AF, enriching the current understanding of how comorbid cardiometabolic pathology intersects with thrombogenic biology in arrhythmic disease.

The CHA_2_DS_2_-VASc score is the main guideline-endorsed tool for thromboembolic risk stratification in AF, with diabetes contributing one point based on its established association with stroke [6]. Nevertheless, its predictive performance has been described as modest, and efforts to enhance risk stratification through biomarkers are ongoing [28]. Our CHA_2_DS_2_-VASc-stratified analyses showed higher D-dimer levels in diabetic patients across all risk categories, linking clinical risk scores to coagulation biology and suggesting that D-dimer may capture residual risk not fully reflected by clinical scoring alone.

### 4.3. Biological Mechanisms Linking Type 2 Diabetes, Atrial Fibrillation, and Coagulation Activation

The association between T2DM and higher D-dimer levels in AF is supported by established mechanisms, as diabetes promotes a prothrombotic state with increased coagulation activation, impaired fibrinolysis, platelet hyperreactivity, and endothelial dysfunction, leading to greater thrombin generation and fibrin turnover reflected by elevated D-dimer [20,22,50,51].

A key driver of this prothrombotic state is increased coagulation factor activity, with T2DM associated with higher fibrinogen, factor VII, factor VIII, and von Willebrand factor levels, indicating enhanced activation of intrinsic and extrinsic pathways [55]. Elevated fibrinogen, in particular, is a consistent finding and contributes to a substrate that favors rapid fibrin formation and subsequent fibrinolysis [50].

Closely related to altered coagulation factor activity is abnormal fibrin clot structure in diabetes. Fibrin polymers formed in hyperglycemic environments tend to be denser, composed of thinner fibers with smaller pore sizes, and more resistant to plasmin-mediated lysis [20,55,56]. These changes predispose to persistent microthrombi and ongoing fibrin degradation, thereby increasing circulating D-dimer levels [50]. Moreover, persistent exposure to hyperglycemia induces glycation of fibrinogen, further modifying fibrin network architecture and contributing to reduced fibrinolytic susceptibility [50].

Impaired fibrinolysis also contributes in T2DM, as elevated PAI-1 inhibits tPA and urokinase, reduces plasmin generation, and slows clot breakdown, promoting persistent coagulation activity and higher fibrin degradation products such as D-dimer [55,57,58].

Endothelial dysfunction is also central to the prothrombotic state associated with diabetes. Endothelial cells normally regulate vascular homeostasis by balancing anticoagulant and procoagulant activities [59,60]. In T2DM, chronic hyperglycemia, oxidative stress, and inflammation impair nitric oxide production and increase expression of adhesion molecules and tissue factor, tipping the balance toward a procoagulant surface that supports thrombin generation and fibrin formation [61,62,63]. These same endothelial disturbances are present in AF, where abnormal flow patterns and atrial remodeling further impair endothelial integrity and promote exposure of procoagulant substrates [64,65]. The superimposition of diabetes-induced endothelial dysfunction on AF-driven endothelial abnormalities likely amplifies systemic coagulation activation.

Platelet hyperreactivity in T2DM is another mechanism contributing to enhanced thrombogenesis. Diabetic platelets exhibit increased activation and aggregation responses, partly due to alterations in intracellular signaling and receptor expression in the setting of insulin resistance [66,67,68]. This behavior increases the interaction between platelets and coagulation factors, promoting thrombus formation and subsequent fibrinolytic activity that generates D-dimer fragments [20,66].

Chronic low-grade inflammation in T2DM further promotes thrombosis, as cytokines such as IL-6 and TNF-α increase acute-phase fibrinogen production, enhance procoagulant protein expression, and reduce endogenous anticoagulant activity [69,70]. These inflammatory changes potentiate both coagulation activation and endothelial dysfunction, linking metabolic derangements with hemostatic imbalance [71].

### 4.4. Clinical Implications and Future Directions

These findings have practical implications for AF care, particularly in patients with T2DM. Higher D-dimer levels suggest residual thromboembolic risk beyond clinical scores, although CHA_2_DS_2_-VASc remains the cornerstone for anticoagulation decisions in non-valvular AF [6,28]. However, several studies highlight limitations of risk scores that rely solely on clinical variables, and growing evidence suggests that integration of circulating biomarkers such as D-dimer can enhance thromboembolic risk prediction [72,73]. For example, the ABC-stroke risk score, which includes biomarkers like natriuretic peptides and troponin, outperforms clinical scores alone, suggesting that biomarkers provide additive prognostic information [8,54].

In our cohort, D-dimer remained higher in diabetic AF patients even after adjustment for age, renal function, heart failure, and CHA_2_DS_2_-VASc score, which is clinically relevant because elevated D-dimer in AF has been linked to higher risks of stroke, systemic embolism, and mortality [38,39]. In a nationwide multicenter cohort of AF patients with stroke, baseline D-dimer predicted recurrent events and helped identify patients who were more likely to benefit from anticoagulants over antiplatelet therapy [74]. Similarly, studies have reported that D-dimer levels correlate with left atrial thrombus and spontaneous echo contrast, markers of thromboembolic propensity in AF, reinforcing its role as an integrative marker of hypercoagulability and embolic potential [39,75].

Compared with other routinely available biomarkers, D-dimer reflects active fibrin turnover rather than upstream inflammatory activation. Markers such as fibrinogen capture procoagulant substrate availability, while CRP reflects systemic inflammation, both of which may also contribute to thrombogenicity in AF. In clinical practice, combined interpretation of coagulation and inflammatory biomarkers may help contextualize residual thrombotic risk.

Oral anticoagulation is the cornerstone of stroke prevention in AF and is recommended above guideline CHA_2_DS_2_-VASc thresholds, with DOACs preferred over VKAs due to more predictable pharmacology and lower intracranial bleeding risk [76,77,78]. This observation aligns with prior work showing that biologic risk (e.g., fibrin turnover markers) may remain elevated even when traditional therapy suppresses clinical events, thereby identifying a subgroup with persistently activated coagulation [79].

Longer duration and greater severity of diabetes are associated with progressively higher thromboembolic risk in AF, underscoring the importance of optimal glycemic control and metabolic risk management even after accounting for anticoagulation and clinical risk factors [40].

Future research should evaluate integrating coagulation biomarkers with clinical scores in prospective cohorts. Combining D-dimer with inflammatory and cardiac stress markers may improve risk discrimination and support more individualized anticoagulation strategies, including precision medicine and machine learning-based models [54,80,81].

From a clinical perspective, these findings suggest that diabetic AF patients may represent a subgroup with persistently increased coagulation activity, even when guideline-directed anticoagulation is applied. While D-dimer is not currently used as a standalone marker to guide anticoagulant initiation or intensification, it may have future value as an adjunct for identifying residual prothrombotic risk in metabolically high-risk AF phenotypes.

### 4.5. Strengths and Limitations

A key strength of the present study is its real-world design with comprehensive phenotyping of patients with atrial fibrillation, enabling a direct and clinically relevant comparison of D-dimer levels between those with and without T2DM. By including a well-matched cohort of approximately equal size for the diabetic and non-diabetic groups, and by applying CHA_2_DS_2_-VASc-stratified analyses, we were able to demonstrate that the association between T2DM and higher D-dimer persists across clinical risk strata and remains robust after multivariable adjustment. This design improves on simpler cross-sectional comparisons that do not account for underlying thromboembolic risk, and it aligns with calls in the literature for biomarker assessment to complement clinical scoring in AF [39].

Another strength is the use of multiple sensitivity analyses, including restriction to patients on oral anticoagulation and exclusion of extreme D-dimer outliers, which show consistent direction and magnitude of effect. This approach enhances confidence that observed differences are not driven by measurement artifacts or baseline imbalance in anticoagulation status.

The study also benefits from contextualizing results within a mechanistically plausible framework. Diabetes is well described as a prothrombotic state with endothelial dysfunction, increased coagulation factor activity, and impaired fibrinolysis, and these processes have been linked to both arterial and venous thrombotic risk [55,58,82].

However, several limitations should be acknowledged. First, the retrospective and observational design prevents causal inference between type 2 diabetes mellitus and D-dimer elevation in atrial fibrillation. Therefore, our findings should be interpreted as an association rather than evidence of a causal pathway, and residual confounding from unmeasured variables cannot be fully excluded.

The retrospective and observational design precludes causal inference, and it remains possible that unmeasured confounders contribute to the observed associations. For example, data on glycemic control (e.g., glycated hemoglobin levels), duration of diabetes, and specific diabetes treatments were not available for every patient and could influence coagulation status. Such clinical details have been shown in prior work to modify fibrinolytic pathways and endothelial function in T2DM and could partially mediate the association we observed [83].

Because this was a single-center cohort from Eastern Europe, external validity may be limited, and replication in multi-center and multi-ethnic populations is warranted.

Because HbA1c and diabetes duration were incompletely available, we could not quantify dose–response relationships between metabolic burden and D-dimer levels.

Although we adjusted for major clinical risk factors, including heart failure and renal function, residual confounding remains a possibility, particularly in relation to inflammation and microvascular complications, which are not fully captured by routine clinical variables. Chronic low-grade inflammation and complement activation have been implicated in both diabetic vasculopathy and enhanced coagulation, suggesting that additional systemic processes may underlie elevated D-dimer in T2DM [84].

A specific methodological limitation concerns the single measurement of D-dimer, which reflects a snapshot in time rather than dynamic changes; repeated measurements might better characterize the temporal relationship between metabolic stress and coagulation activity. Additionally, D-dimer assays vary by method and reference range, and although our study used a consistent institutional assay, external validity to other settings with different assays may be limited.

Furthermore, while we endeavored to exclude patients with acute thrombotic or inflammatory conditions that would markedly elevate D-dimer, it remains possible that subclinical events could influence results. This is a common challenge in biomarker studies—distinguishing between chronic baseline activation and acute perturbations—and highlights the importance of correlating biomarker levels with clinical outcomes in prospective cohorts.

Finally, this is a single-center study, which may limit generalizability. Differences in population characteristics, healthcare systems, and laboratory practices could influence the applicability of our findings to broader or more diverse AF populations. Future multi-center studies are needed to confirm these results across geographic and ethnic groups.

## 5. Conclusions

In this real-world cohort of patients with atrial fibrillation, type 2 diabetes mellitus was consistently associated with higher plasma D-dimer levels, indicating increased coagulation activity. This association persisted across clinically relevant D-dimer thresholds, within CHA_2_DS_2_-VASc risk categories, and after multivariable adjustment and sensitivity analyses, suggesting a more pronounced hypercoagulable phenotype in diabetic AF patients beyond clinical risk stratification alone.

The persistence of higher D-dimer levels in patients with diabetes, including those receiving oral anticoagulation, provides biological context for the elevated thromboembolic risk observed in this population and supports the concept of residual hypercoagulability in AF with T2DM. These findings highlight the potential role of coagulation biomarkers in refining thromboembolic risk assessment, particularly in high-risk metabolic subgroups.

Future prospective studies are warranted to determine whether integrating biomarkers such as D-dimer into clinical risk models can improve individualized risk stratification or inform targeted therapeutic strategies in atrial fibrillation.

## Figures and Tables

**Table 1:** Baseline characteristics of patients with atrial fibrillation according to diabetes status.

Variable	AF + T2DM (*n* = 150)	AF Without T2DM (*n* = 150)	*p*-Value
Age, years (mean ± SD)	72.6 ± 8.9	68.1 ± 10.4	<0.001
Female sex, *n* (%)	70 (46.7)	72 (48.0)	0.82
Hypertension, *n* (%)	129 (86.0)	103 (68.7)	<0.001
Heart failure, *n* (%)	67 (44.7)	44 (29.3)	0.006
Coronary artery disease, *n* (%)	58 (38.7)	45 (30.0)	0.12
Prior myocardial infarction, *n* (%)	29 (19.3)	23 (15.3)	0.36
Prior stroke/TIA, *n* (%)	33 (22.0)	21 (14.0)	0.08
Peripheral arterial disease, *n* (%)	26 (17.3)	15 (10.0)	0.07
Chronic kidney disease (eGFR < 60), *n* (%)	57 (38.0)	32 (21.3)	0.002
Creatinine, mg/dL (median (IQR))	1.14 (0.96–1.38)	1.02 (0.88–1.21)	0.004
eGFR, mL/min/1.73 m^2^ (median (IQR))	61 (48–75)	72 (58–86)	<0.001
CHA_2_DS_2_-VASc score (median (IQR))	4 (3–5)	3 (2–4)	<0.001
HAS-BLED score (median (IQR))	2 (1–3)	2 (1,2)	0.09
Oral anticoagulation, *n* (%)	124 (82.7)	120 (80.0)	0.56
Direct oral anticoagulant, *n* (%)	92 (61.3)	95 (63.3)	0.72
Vitamin K antagonist, *n* (%)	32 (21.3)	25 (16.7)	0.32
No anticoagulation, *n* (%)	26 (17.3)	30 (20.0)	0.56

Baseline demographic and clinical characteristics of patients with atrial fibrillation stratified by type 2 diabetes mellitus status.

**Table 2:** D-dimer levels according to diabetes status.

Variable	AF + T2DM (*n* = 150)	AF Without T2DM (*n* = 150)	*p*-Value
D-dimer, µg/mL FEU (median (IQR))	0.94 (0.56–1.62)	0.63 (0.38–1.05)	<0.001
log_10_(D-dimer) (mean ± SD)	−0.03 ± 0.48	−0.20 ± 0.44	<0.001
D-dimer > 0.5 µg/mL FEU, n (%)	111 (74.0)	85 (56.7)	0.002
D-dimer > 1.0 µg/mL FEU, n (%)	64 (42.7)	39 (26.0)	0.003

Comparison of plasma D-dimer levels and categorical D-dimer thresholds between atrial fibrillation patients with and without type 2 diabetes mellitus.

**Table 3:** Multivariable regression analyses for the association between type 2 diabetes mellitus and elevated D-dimer levels.

Model	Outcome	Main Predictor	Adjusted Effect	95% CI	*p*-Value
Linear regression	log_10_(D-dimer)	T2DM (yes vs. no)	β = 0.19	0.09–0.29	<0.001
Logistic regression	D-dimer > 0.5 µg/mL FEU	T2DM (yes vs. no)	OR = 2.18	1.34–3.55	0.002
Logistic regression	D-dimer > 1.0 µg/mL FEU	T2DM (yes vs. no)	OR = 2.07	1.24–3.47	0.005

Multivariable linear and logistic regression models evaluating the independent association between type 2 diabetes mellitus and D-dimer levels in patients with atrial fibrillation. Covariates included age, sex, heart failure, chronic kidney disease, prior stroke/TIA, CHA_2_DS_2_-VASc score, and oral anticoagulation at the time of sampling.

## Data Availability

The original contributions presented in this study are included in the article. Further inquiries can be directed to the corresponding authors.

## Abbreviations

The following abbreviations are used in this manuscript:

AF	Atrial fibrillation
BMI	Body mass index
CHA_2_DS_2_-VASc	Congestive heart failure, Hypertension, Age ≥ 75 years, Diabetes mellitus, prior Stroke or transient ischemic attack, Vascular disease, Age 65–74 years, Sex category
CKD	Chronic kidney disease
DOAC	Direct oral anticoagulant
FEU	Fibrinogen equivalent units
GDPR	General Data Protection Regulation
HbA1c	Glycated hemoglobin
HF	Heart failure
IQR	Interquartile range
DOAC	Non-vitamin K antagonist oral anticoagulant
OAC	Oral anticoagulant
OR	Odds ratio
PAI-1	Plasminogen activator inhibitor-1
SD	Standard deviation
T2DM	Type 2 diabetes mellitus
VKA	Vitamin K antagonist
